# Adenovector 26 Encoded RSV Prefusion F Protein (Ad26.RSV.preF) Does Not Predispose to Enhanced Respiratory Disease in Preclinical Rodent Models

**DOI:** 10.3390/vaccines14010087

**Published:** 2026-01-15

**Authors:** Renske Bolder, Susan B. S. King, Roland C. Zahn, Leslie van der Fits

**Affiliations:** Johnson & Johnson Innovative Medicine, 2333 CN Leiden, The Netherlands

**Keywords:** respiratory syncytial virus, adenoviral vector 26, RSV prefusion protein, enhanced respiratory disease

## Abstract

Background/objectives: RSV is a major cause of mortality in infants, and despite recent progress to prevent RSV in the very young, an RSV vaccine for this population is still highly warranted. Clinical studies in infants in the 1960s using formalin-inactivated RSV (FI-RSV) led to life-threatening enhanced respiratory disease (ERD). Therefore, a thorough safety assessment of RSV vaccine candidates intended for RSV seronegative infants is crucial. Methods: Prior to clinical pediatric development of Ad26.RSV.preF, an adenovirus type 26 vector-encoding RSV F protein stabilized in its prefusion conformation, predisposition to ERD was extensively assessed in cotton rat and mouse models. Results: Cotton rats intramuscularly immunized with a wide dose range of Ad26.RSV.preF, including low and sub-protective vaccine doses, and challenged with vaccine homologous RSV A2 or heterologous RSV B Wash 18537, did not show signs of predisposition to ERD. Histopathology scores for alveolitis, peribronchiolitis, interstitial pneumonia, and perivasculitis after challenge were significantly lower for Ad26.RSV.preF-immunized cotton rats compared to FI-RSV-immunized cotton rats and comparable to or lower than scores in cotton rats intranasally pre-exposed to RSV prior to challenge to mimic natural repeated infection. These results were observed in animals with or without viral replication in the lung after RSV challenge, in the presence or absence of vaccine-induced antibodies. Similar results were observed in mice, where more extensive assessment of mono- and polymorphonuclear cell alveolitis, mucus cell hyperplasia, and mucus accumulation was performed. Conclusions: Based on these extensive analyses, we conclude that there are no indications of ERD predisposition after Ad26.RSV.preF vaccination in rodent models, irrespective of the vaccine dose, challenge virus strain, or presence of viral replication in the lung. These results are crucial for the pediatric development of this vaccine.

## 1. Introduction

Respiratory syncytial virus (RSV) is currently the major cause of severe acute respiratory disease in children under five years of age, with a global estimation of 3.6 million hospitalizations and over 100.000 deaths in 2019 [1]. There is also growing evidence to suggest that RSV lower respiratory tract infections in early childhood are associated with subsequent long-term wheezing, asthma, and impaired lung function [2]. In the past years, major steps have been taken to protect the most vulnerable population of children from birth up to 6 months of age with the development of a long-acting human monoclonal antibody directed against prefusion F protein (preF) that requires a single intramuscular dose to protect neonates and infants from lower respiratory tract disease (LRTD) during their first RSV season [3]. In addition, a maternal preF protein vaccine was licensed, which can protect infants up to 6 months of age against LRTD when given during pregnancy (24–36 weeks of gestation) [4]. While this passive immunity can protect infants during their first RSV season, there is currently no licensed vaccine available for infants that induces active immunity [5]. In contrast to these prophylactic interventions, active immunization could provide long-lasting protection and can be a valuable addition in resource-limited settings.

Development of an RSV vaccine for the RSV-seronegative pediatric population had been greatly impeded by the legacy of vaccine-induced enhanced respiratory disease (ERD) that was observed in clinical studies in the 1960s conducted in infants and children immunized with formalin-inactivated RSV (FI-RSV). FI-RSV-vaccinated infants were hospitalized at a higher rate and with more severe disease after RSV infection compared with unvaccinated infants, with two FI-RSV-vaccinated infants dying from respiratory disease. In the lungs of the deceased infants with ERD, excessive infiltration of inflammatory cells, including mononuclear cells, eosinophils, and polymorphonuclear cells, was observed [6,7,8]. Although the underlying mechanisms of ERD are not fully clarified, studies in animal models have shown that important factors associated with FI-RSV-induced ERD included the induction of poorly RSV-neutralizing antibodies, a Th2-skewed CD4+ response lacking cytotoxic CD8+ T cell priming, and infiltration of inflammatory cells into the lungs after RSV challenge [9,10,11,12].

Several animal models have been described for ERD assessment. Applying FI-RSV and subsequent RSV infection in animals can induce similar immunopathology as that of the deceased infants with ERD [12]. The cotton rat (*Sigmodon hispidus*) is a widely used rodent model for RSV vaccine evaluation and ERD assessment. Cotton rats immunized with FI-RSV and subsequently infected with RSV show increased severity of peribronchiolitis, neutrophilic alveolitis, and interstitial pneumonia compared with mock-vaccinated animals. Especially, alveolitis and interstitial pneumonia appeared to be specific markers for the vaccine-induced disease enhancement [13]. In the mouse model, additional ERD markers are described, including mucus hypersecretion and lung eosinophilia. However, the predictive potential of these animal models has not been fully confirmed, other than for FI-RSV, as limited information from human studies in the target population is available.

The degree of risk for predisposition to ERD is likely dependent on the antigen and vaccine platform applied and related to the type and magnitude of the immune response induced by the vaccine. Protein-based approaches can be considered risky due to the lack of MHC class I presentation needed for activation of CD8+ T cells, and RSV F-based subunit soluble vaccines were shown to induce ERD-like symptoms in cotton rats [14]. In contrast, the processing of viral vector-based vaccines and live attenuated vaccines probably makes these platforms most suitable for RSV vaccine development for young infants, as these may efficiently induce CD8+ T-cells and a Th1-associated immune response [15]. The vaccine candidate Ad26.RSV.preF is a recombinant, replication-incompetent adenovirus type 26 (Ad26) vector encoding a membrane-bound full-length F protein from the RSV A2 strain stabilized in its prefusion conformation (preF) using amino acid substitutions that were described earlier [16]. We have previously shown that this vector induced Th1-biased immunity, with long-lasting CD8+ T-cell responses and potent neutralizing antibodies in a neonatal Th2-prone mouse model [17]. Clinical studies have shown that Ad26.RSV.preF is safe, immunogenic, and efficacious in adults [18,19]. In addition, the vector was safe and immunogenic in small cohorts of RSV-seropositive and seronegative toddlers of 12–24 months of age [20,21].

Here we present results of a series of cotton rat and mouse studies to assess predisposition to ERD of the Ad26.RSV.preF vaccine that were executed to de-risk the clinical development of this vector for use in infants. After immunization with Ad26.RSV.preF, there was no indication of vaccine-induced predisposition to ERD, irrespective of the vaccine dose, RSV strain used for challenge, or the level of protective efficacy induced.

## 2. Materials and Methods

### 2.1. Ethics Statement

All animal studies were conducted at Sigmovir Biosystems, Inc., Rockville, MD, USA, by permission of the Institutional Animal Care and Use Committee (IACUC) of Sigmovir Biosystems, Inc.

### 2.2. Animals and Animal Handling

Female cotton rats (Sigmovir Biosystems, Inc.; 6–8 weeks old) or mice (Envigo, Indianapolis, IN, USA; 6–8 weeks old) were immunized intramuscularly at Week 0 with formulation buffer (mock), the indicated doses of Ad26.RSV.preF [22], or at Week 0 and Week 4 with FI-RSV, for which the original lot 100 that induced ERD in clinical studies [7] was used in the same dilution that was shown to induce ERD in cotton rats (1:125) [13]. A control group received 10^4^ pfu live RSV A2 (VR-1540, ATCC, Manassas, VA, USA) or RSV B Wash 18537 (Sigmovir, Biosystems, Inc.) intranasally at Week 0. Serum samples were collected at Week 7, prior to challenge, for ELISA or VNAs. At Week 7, animals were challenged intranasally with 10^5^ pfu live RSV A2 or RSV B Wash 18537, or with another dose where indicated. Animals were sacrificed 5 days post-challenge. The left lobe was weighed and homogenized for plaque assay, the right lobe was inflated and fixed in formalin for histopathology, and the lingular lobe was designated for RT-qPCR analysis (cotton rats only). Nasal turbinates were isolated, estimated at 0.3 g, and homogenized for plaque assay.

### 2.3. Viral Load Determination

#### 2.3.1. By Plaque Assay

Viral load was determined on lung and nose homogenates by plaque titration, performed in duplicate on HEp-2 cells (CCL-23, ATCC) in a 24-well format. Cells and homogenates were incubated for 2 h, supernatant was aspirated, and wells were overlayed with methylcellulose media until plaque formation (4 to 7 days, depending on the RSV strain used). Plates were stained with crystal violet, and plaques were counted to determine the virus concentration in pfu/g of tissue.

#### 2.3.2. By Reverse Transcription-Quantitative Polymerase Chain Reaction (RT-qPCR)

RT-qPCR was performed essentially according to Boukhvalova et al. [23]. Total RNA was extracted from snap-frozen lingular lung lobes and used to generate cDNA. Real-time PCR was performed for the RSV NS1 gene (primer sequences CACAACAATGCCAGTGCTACAA and TTAGACCATTAGGTTGAGAGCAATGT) and beta-actin (primer sequences TACGCCAACACAGTGCTGTCT and TCTGCATCCTGTCGGCAAT) using iQ^TM^ SYBR Green Supermix (BioRad, Hercules, CA, USA) on a iCycler (BioRad). Cycle thresholds (Ct) were calculated by the iQ5 software and converted to relative units using a standard curve. These relative units were then normalized to the level of beta-actin mRNA (“housekeeping gene”) expressed in the corresponding sample.

### 2.4. Histopathology

Histopathology was performed on lungs from cotton rats and mice, similarly to previously described [13]. Lung tissue fixed in formalin was embedded in paraffin, cut in 5-μm sections, deparaffinized, and stained with hematoxylin and eosin (H&E). Slides were blinded and examined microscopically. Pulmonary inflammation was evaluated by scoring the following parameters: alveolitis (cells within the alveolar spaces), interstitial pneumonia (inflammatory cell infiltration and thickening of alveolar walls), peribronchiolitis (inflammatory cell infiltration around the bronchioles), and perivasculitis (inflammatory cell infiltration around the small blood vessels). Slides were scored blindly on a 0–4 severity scale, being: Score 0: No lesions; Score 1: Up to 5% of lung is compromised with severe lesions, or up to 25% with mild lesions; Score 2: Up to 25% of lung is compromised with severe lesions, or up to 75% with mild lesions; Score 3: Up to 75% of lung is compromised with severe lesions, or up to 100% with mild lesions; Score 4: Up to 100% of lung is compromised with severe lesions.

In addition, serial sections of the paraffin-embedded mouse lungs were stained with alcian blue (pH 2.5) and periodic acid-Schiff (PAS) for conducting analysis of airway cells containing mucosubstances. The severity and distribution of mucous cell hyperplasia/hypertrophy were both graded on the scale from 0 to 4, and scores depicted represent the product of severity and distribution scores. Similarly, bronchiolar luminal containment of mucus, exudates of inflammatory cells, and cell debris was also graded subjectively with similar distribution and severity scores, and the product of these was calculated and indicated as the luminal mucus accumulation score.

### 2.5. RSV F Enzyme-Linked Immunosorbent Assay (ELISA)

For cotton rat samples, RSV F binding IgG antibodies were measured by ELISA, using RSV F protein (wild type, postF conformation) as coating antigen, followed by serially diluted serum. Cotton rat RSV F-specific antibodies were detected by HPR-labeled anti-cotton rat IgG (ICL Inc., Portland, OR, USA), and wells were developed with OPD substrate (Thermo Fisher Scientific, Waltham, MA, USA). Endpoint titers were calculated by linear interpolation.

For mouse samples, IgG antibodies to RSV F were measured by ELISA using streptavidin-coated plates incubated with biotin-labeled F protein (wild type, postF conformation), followed by serially diluted serum. Mouse RSV F-specific antibodies were detected by HRP-labeled anti-mouse IgG (BioRad), and the wells were developed with LumiGlo (SeraCare, Milford, MA, USA). The luminescence signal was measured, and relative potency against a standard sample was calculated.

### 2.6. Virus Neutralization Assay (VNA)

VNA titers against RSV clinical isolate CL57 were determined in mouse or cotton rat serum samples using a recombinant RSV encoding firefly luciferase (FFL), harboring the G and F genes of RSV strain CL57 in an RSV A2 backbone, that was derived via a BAC-based reverse genetics system as described in Hotard et al. [24]. Serial dilutions of heat-inactivated sera were mixed with RSV CL57 FFL and incubated prior to the addition of A549 cells (CCL-185, ATCC). After an incubation period of 20 h, neolite substrate (Perkin Elmer, Waltham, MA, USA) was added, and the luminescence signal was measured. VNA titers were calculated as the antibody concentration that caused a 90% reduction in luminescence (expressed as IC90 values (log2)).

### 2.7. Data and Statistical Analysis

Power calculations were performed to determine the group sizes, with the histopathological alveolitis score as the primary outcome parameter.

Animal studies were considered valid if there was a significant reduction in RSV titers observed in the lungs of challenged animals that were mock immunized versus animals that received i.n. pre-exposure to RSV based on a Wilcoxon rank-sum test. In addition, analysis of ERD in each study was considered valid if a significant difference was observed in alveolitis scores between the negative control groups (mock immunized and/or intranasal pre-exposure to RSV) and the positive control group (FI-RSV) based on a Wilcoxon rank-sum test. Virological or immunological readouts were considered valid if the appropriate technical controls taken along during the individual assays were within pre-defined specifications. Only studies and data points that fulfilled those criteria were included in the manuscript.

Statistical testing of ERD outcome parameters was performed by comparing Ad26.RSV.preF-immunized (sub)groups with the positive and negative ERD controls, i.e., FI-RSV and mock-immunized or RSV intranasal pre-exposed animals, respectively, using the non-parametric Wilcoxon rank-sum test, with Bonferroni correction for multiple testing when more than one Ad26.RSV.preF-immunized (sub)group was compared.

Statistical testing of immunogenicity and efficacy readouts was performed by comparing the Ad26.RSV.preF-immunized groups with the mock-immunized control group, using the non-parametric Wilcoxon rank-sum test with Bonferroni correction or, if most measurements were above LOD/LLOQ, using ANOVA for potentially censored values with Bonferroni or Dunnett correction.

Statistical analysis was performed using GraphPad Prism or SAS.

## 3. Results

### 3.1. No Histopathological Signs of Enhanced Respiratory Disease Observed in Ad26.RSV.preF-Immunized Cotton Rats After Challenge with RSV A2

We have shown previously that a single intramuscular immunization with 10^5^ to 10^8^ viral particles (vp) of Ad26.RSV.preF dose-dependently induces neutralizing antibodies in naïve cotton rats and protects against viral replication after challenge with RSV A2, with complete absence of replicating virus in the lungs of animals that seroconverted after vaccination [22].

Predisposition to ERD after immunization with 10^5^ to 10^8^ vp Ad26.RSV.preF and subsequent RSV challenge was assessed by histopathological analysis in multiple cotton rat studies. FI-RSV was given twice as a positive control for the induction of ERD, whereas negative ERD controls included a mock-immunized group, as well as a group that received an intranasal RSV A2 infection prior to challenge in order to mimic natural repeated exposure. Additionally, naïve, untreated, and unchallenged animals were included to serve as baseline controls. Representative examples of histopathological pictures of the different (control) groups are shown in Figure S1.

**Figure 1:**
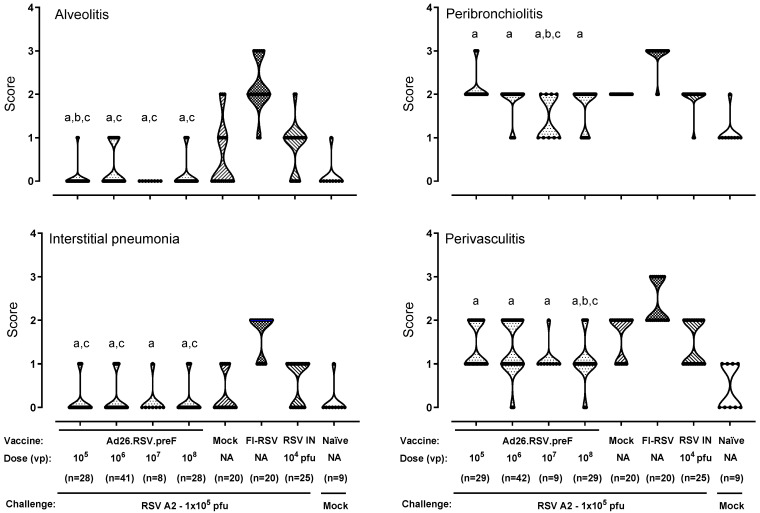
Ad26.RSV.preF does not predispose for ERD in the cotton rat RSV A2 challenge model. Cotton rats were immunized with Ad26.RSV.preF, in a dose range from 10^5^ to 10^8^ vp, whereas control groups received formulation buffer (mock), FI-RSV, or live RSV A2 intranasally (RSV IN). At 49 days post-immunization, animals were intranasally challenged with RSV A2 (1 × 10^5^ pfu). Naïve mock challenged cotton rats are included as baseline control, indicated as “Naïve”. H&E-stained sections from lung tissue isolated at day 5 post challenge were scored for alveolitis, peribronchiolitis, interstitial pneumonia, and perivasculitis on a scoring scale from 0 to 4. Scores of individual animals are shown with black dots, and the frequency distribution is indicated by the shape of the violin plot. Results from statistical analysis comparing the scores of the Ad26.RSV.preF-immunized groups with the FI-RSV, mock-immunized, or RSV intranasally pre-exposed control groups are indicated with a, b, or c, respectively, where *p* ≤ 0.05. Results shown are a compilation of 3 independent studies using identical study setups. NA: not applicable.

As expected, the FI-RSV-vaccinated animals all showed positive alveolitis scores varying between 1 and 3, with a median score of 2 (Figure 1). For the mock-immunized and RSV-pre-exposed animals, the individual alveolitis scores varied between 0 and 2, with a median response of 0 and 1, respectively. The majority of the Ad26.RSV.preF-immunized animals (86 out of 105) showed no alveolitis lesions (score 0), and only a very few animals had a score of 1. Scores seemed unrelated to the vaccine dose. These low alveolitis scores were significantly lower compared to the FI-RSV control (*p* < 0.0004) and RSV intranasal pre-exposed group (*p* ≤ 0.0044) (Wilcoxon rank-sum test with 4-fold Bonferroni correction) and similar or significantly lower than scores observed in the mock immunized control group.

For the other inflammatory parameters, FI-RSV-immunized animals also showed mild to severe lesions with median scores of 2 for interstitial pneumonia and perivasculitis and a median score of 3 for peribronchiolitis. For the mock- and RSV-pre-exposed animals, scores for these inflammatory parameters were lower compared to the FI-RSV-immunized animals. For the Ad26.RSV.preF-immunized animals, the inflammatory scores were significantly lower compared to the FI-RSV control (*p* < 0.0004) (Wilcoxon rank-sum test with 4-fold Bonferroni correction) and similar to or significantly lower than scores observed in the mock or RSV pre-exposed control animals (Figure 1). Altogether, this histopathological analysis of over 100 Ad26.RSV.preF-immunized cotton rats did not give any indication of predisposition to ERD by Ad26.RSV.preF immunization.

### 3.2. No Histopathological Alterations Characteristic for ERD Are Observed in Ad26.RSV.preF-Immunized Animals with Detectable RSV A2 Lung Viral Load After Challenge

It is postulated that vaccine-associated ERD in the cotton rat model is most likely to be ascertained when animals show adequate viral replication following RSV challenge in the presence of vaccine-induced antibodies [15]. Based on these criteria, only a single animal out of the more than one hundred cotton rats challenged with 10^5^ pfu RSV A2 that are displayed in Figure 1 could be considered the most relevant for ERD assessment. All other animals either had no detectable antibodies indicative of vaccine take or showed no lung viral load by plaque assay. We therefore applied several strategies to generate a more stringent challenge model that would result in animals with detectable antibody titers as well as detectable lung viral load in order to perform a more thorough ERD assessment.

Firstly, the RSV A2 challenge dose was increased from 1 × 10^5^ pfu to 5 × 10^5^ or 1 × 10^6^ pfu, in combination with low vaccine doses of Ad26.RSV.preF (2 × 10^5^ to 1 × 10^6^ vp). The increase in challenge dose did not enhance the stringency of the RSV A2 challenge, as all animals with detectable antibodies still had undetectable viral load in the lung, except for a single animal with low lung viral load (Figure S2A). Alveolitis scores showed a trend toward increased histopathology at higher challenge virus doses in both vaccinated and control groups (Figure S2B). For Ad26.RSV.preF-immunized cotton rats, statistical analysis showed that alveolitis scores across challenge doses were lower compared with the FI-RSV group for all Ad26.RSV.preF vaccine doses and not different from the mock and RSV IN control groups (Cochran–Mantel–Haenszel test with Bonferroni correction for multiple comparisons).

**Figure 2:**
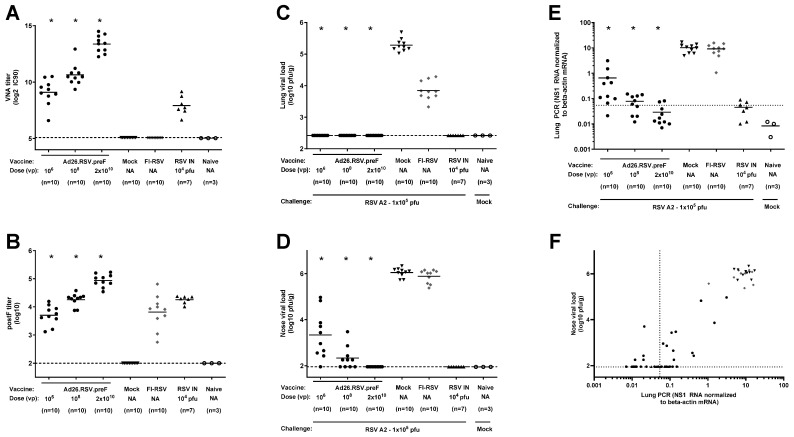
Ad26.RSV.preF induces protective immunity in RSV A2-challenged cotton rats, whereas vaccine-dose-dependent lung viral load can be detected by RT-qPCR. Cotton rats were immunized once with 10^6^ or 10^8^ vp Ad26.RSV.preF at day 0 or twice with 2 × 10^10^ vp at days 0 and 28, whereas control groups received formulation buffer (mock), FI-RSV, or live RSV intranasally (RSV IN). Serum collected at day 49 was used to determine VNA titers against RSV CL57 (**A**) or F-binding antibody titers (**B**). On day 49, animals were intranasally challenged with RSV A2 (1 × 10^5^ pfu). Naïve mock-challenged cotton rats are included as baseline control, indicated as “Naïve”. Five days post-challenge, RSV viral load was determined in homogenates of lung (**C**) and nasal turbinates (**D**) by plaque assay. (**E**) RSV viral load was determined in lung homogenates by determining RSV NS1 RNA expression by RT-qPCR. A very conservative threshold for defining positivity was defined as geomean + 10× standard deviation of naïve mock-challenged animals and indicated with a dotted line. (**F**) Correlation plot of lung RSV viral load as determined by RT-qPCR with nose viral load as determined by plaque assay. Means are indicated with horizontal lines, and LOD or LLoQ of the assays are indicated with dashed lines. Significant differences (adjusted *p* ≤ 0.05) between Ad26.RSV.preF and mock-immunized groups are indicated with asterisks.

Secondly, a more sensitive method for the detection of lung viral load was applied by performing RT-qPCR for the RSV A2 NS1 gene in lung homogenates. Cotton rats were immunized once with 10^6^ or 10^8^ vp Ad26.RSV.preF at day 0 or twice with 2 × 10^10^ vp at days 0 and 28. All animals showed detectable RSV-neutralizing and F-binding antibodies prior to challenge at day 49 (Figure 2A,B) that were significantly higher compared to the mock-immunized animals. Immunization with FI-RSV induced RSV F binding antibodies lacking neutralizing capacity, whereas the intranasal RSV pre-exposed control group showed F binding antibody as well as neutralizing antibody titers. At day 5 post-challenge, lung and nose viral load was determined by plaque assay. As expected, no lung viral load could be detected for the Ad26.RSV.preF-immunized animals and RSV-pre-exposed animals, while the mock- and FI-RSV-immunized animals all showed lung viral load (Figure 2C). The nose viral load was significantly reduced for the Ad26.RSV.preF-immunized cotton rats and below detection for the animals that received two doses of 2 × 10^10^ vp (Figure 2D). Use of RT-qPCR for the detection of RSV viral load in lung homogenates appeared more sensitive compared to the plaque assay. The Ad26.RSV.preF-immunized animals showed a dose-dependent significant reduction in the lung viral load as determined by RT-qPCR, which was lower than the viral load in the mock-immunized animals (Figure 2E). When applying a conservative threshold for defining positivity based on the signal in the mock-challenged animals, 9/10, 6/10, and 2/10 animals with positive viral load were identified in the 10^6^ vp, 10^8^ vp, and 2 × 10^10^ vp dose groups, respectively. The lung viral load detected by RT-qPCR correlated well with the nose viral load detected by plaque assay (*p* < 0.0001; Spearman rho = 0.8417) (Figure 2F).

**Figure 3:**
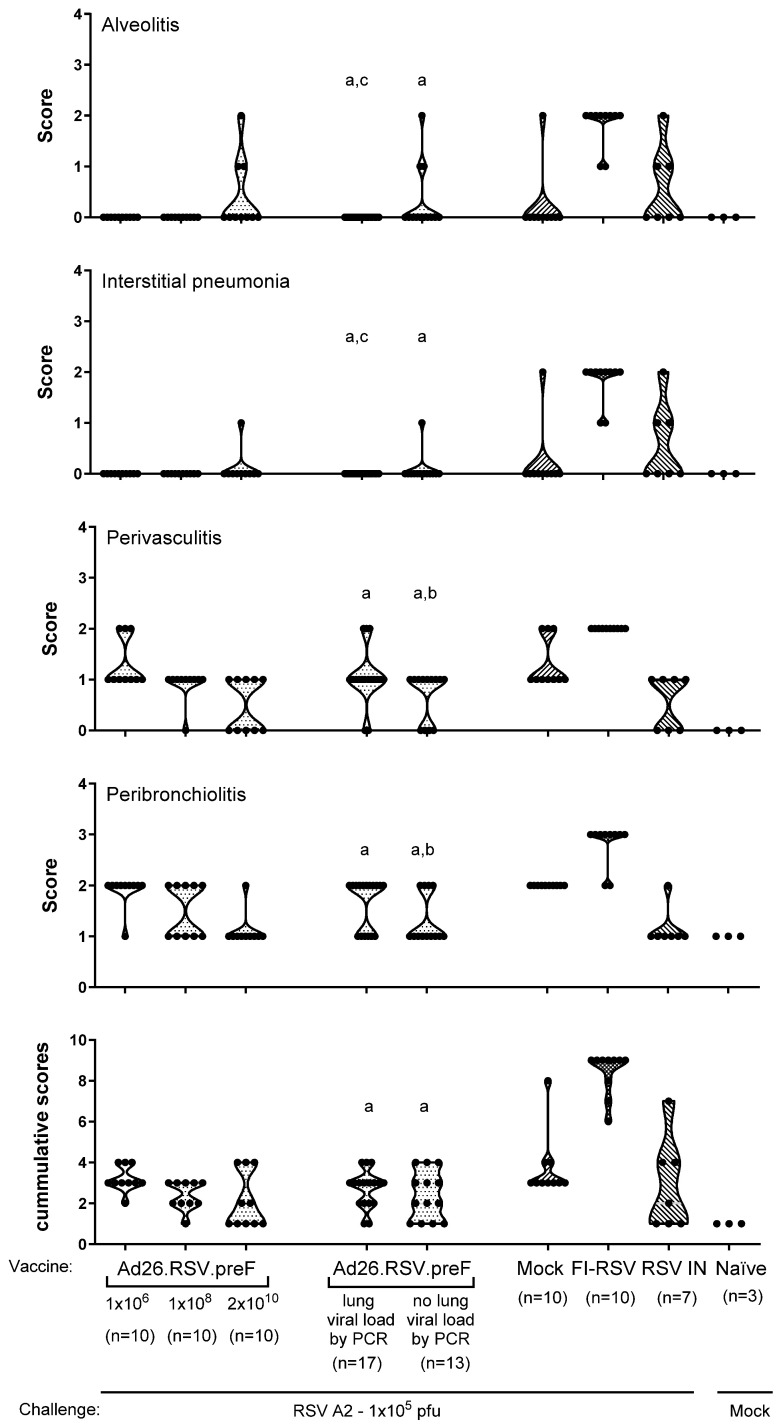
Ad26.RSV.preF does not induce histopathological alterations characteristic of ERD after RSV A2 challenge in cotton rats, irrespective of detectable lung viral load. Cotton rats were immunized once with 10^6^ or 10^8^ vp Ad26.RSV.preF at day 0 or twice with 2 × 10^10^ vp at days 0 and 28, whereas control groups received formulation buffer (mock), FI-RSV, or live RSV intranasally (RSV IN). On day 49, animals were intranasally challenged with RSV A2 (1 × 10^5^ pfu). Naïve mock-challenged cotton rats are included as baseline control, indicated as “Naïve”. At 5 days post-challenge, histopathology scores for alveolitis, interstitial pneumonia, peribronchiolitis, and perivasculitis were determined in H&E-stained lung sections of the different groups. In addition, these same animals that were immunized with different doses of Ad26.RSV.preF were stratified based on the presence of RSV NS1 RNA in the lung, as defined using a conservative threshold of geomean +10× standard deviation of mock-challenged animals, and these are additionally depicted as separate groups. Cumulative scores (bottom panel) were calculated as the sum of the different histopathology scores. Scores of individual animals are shown with black dots, and the frequency distribution is indicated by the shape of the violin plot. Results from statistical analysis comparing the scores of the Ad26.RSV.preF-immunized groups with the FI-RSV, mock-immunized, or RSV intranasally pre-exposed control groups are indicated with a, b, or c, respectively, where *p* ≤ 0.05.

Lung sections of animals immunized with 10^6^, 10^8^, or 2 × 10^10^ vp Ad26.RSV.preF were examined. Histopathology scores were lower than the scores in FI-RSV-immunized animals and similar to or lower than control groups that received mock immunization or RSV intranasal pre-exposure (Figure 3, not statistically tested). In addition, lung histopathology scores were separately assessed in subgroups of these same Ad26.RSV.preF-immunized animals stratified for the presence (*n* = 17) or absence of lung viral load (*n* = 13) detected by RT-qPCR. For all four parameters of pulmonary inflammation, scores for the Ad26.RSV.preF-immunized cotton rats with or without RT-qPCR-detectable viral load were significantly lower compared with the FI-RSV control (*p* < 0.0002) and not significantly different, or significantly lower in some cases, from the mock-immunized or RSV intranasal pre-exposed control groups (Figure 3) (Wilcoxon Rank Sum test with 2-fold Bonferroni correction). Cumulative scores (Figure 3, bottom panel) were calculated by combining the scores of the four parameters. For all Ad26.RSV.preF-immunized animals, both with and without lung viral load by RT-qPCR, cumulative inflammation scores were not significantly different from the mock and RSV intranasal pre-exposed cotton rats and were significantly lower compared to FI-RSV-immunized animals (*p* < 0.0002, Wilcoxon Rank Sum test with 2-fold Bonferroni correction). These data demonstrate that there is no indication of ERD induction in RSV A2-challenged cotton rats after immunization with Ad26.RSV.preF, irrespective of the presence of detectable viral load in the lung.

### 3.3. After Challenge with Vaccine Heterologous RSV B Wash 18537, No Signs of Enhanced Respiratory Disease Were Observed in Ad26.RSV.preF-Immunized Cotton Rats

**Figure 4:**
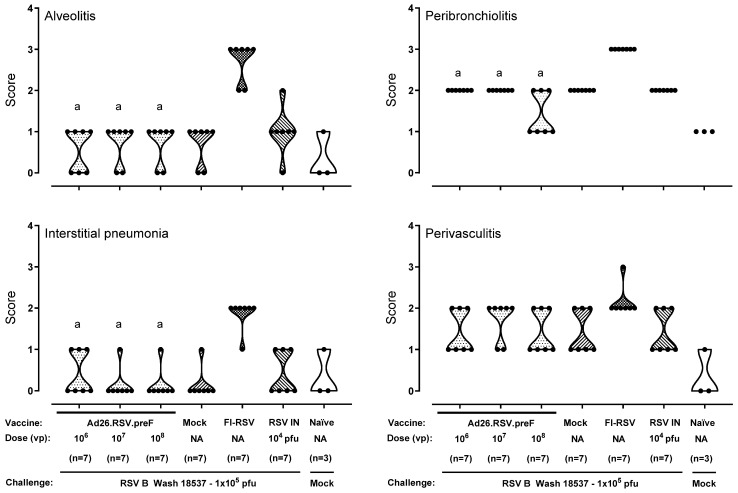
No histopathological signs for ERD predisposition were observed after Ad26.RSV.preF immunization in the cotton rat RSV B Wash challenge model. Cotton rats were immunized with Ad26.RSV.preF in a dose range from 10^6^ to 10^8^ vp, whereas control groups received formulation buffer (mock), FI-RSV, or live RSV B Wash intranasally (RSV IN). At 49 days post-immunization, animals were intranasally challenged with RSV B Wash 18537 (1 × 10^5^ pfu). Naïve mock-challenged cotton rats were included as a baseline control, indicated as “Naïve”. H&E-stained sections made from lung tissue isolated at day 5 post challenge were scored for alveolitis, peribronchiolitis, interstitial pneumonia, and perivasculitis on a scoring scale from 0 to 4. Scores of individual animals are shown with black dots, and the frequency distribution is indicated by the shape of the violin plot. Results from statistical analysis comparing the scores of the Ad26.RSV.preF-immunized groups with the FI-RSV, mock-immunized, or RSV intranasally pre-exposed control groups are indicated with a, b, or c, respectively, where *p* ≤ 0.05. NA: not applicable.

As a third approach for a thorough ERD assessment in the cotton rat model (i.e., in animals with detectable antibody titers as well as detectable lung viral load), histopathological analysis was performed on cotton rats challenged with a vaccine heterologous RSV B Wash 18537 strain. We recently showed the ability of 10^6^ to 10^9^ vp Ad26.RSV.preF to induce cross-protection against challenge with 1 × 10^5^ pfu RSV B Wash 18537 in cotton rats [25]. Heterologous challenge with B Wash was shown to be a more stringent model than a vaccine homologous RSV A2 challenge, which was mainly apparent for nasal viral replication. Histopathological analysis of lung sections of RSV B Wash-challenged animals after vaccination with 10^6^, 10^7^, or 10^8^ vp Ad26.RSV.preF demonstrated significantly lower scores for the parameters assessed when compared to the FI-RSV group (*p* ≤ 0.0069, Wilcoxon rank-sum test with 3-fold Bonferroni correction), except for perivasculitis, which failed to reach significance. In contrast, scores for Ad26.RSV.preF-immunized animals were not significantly different from the mock-vaccinated or intranasal RSV B Wash pre-exposed animals (Wilcoxon rank-sum test with 3-fold Bonferroni correction) (Figure 4). It should be noted, however, that in this study only a single animal of the 10^6^ vp Ad26.RSV.preF group fulfilled the criteria to allow full ERD assessment, i.e., having both detectable antibody titers and detectable lung viral load.

**Figure 5:**
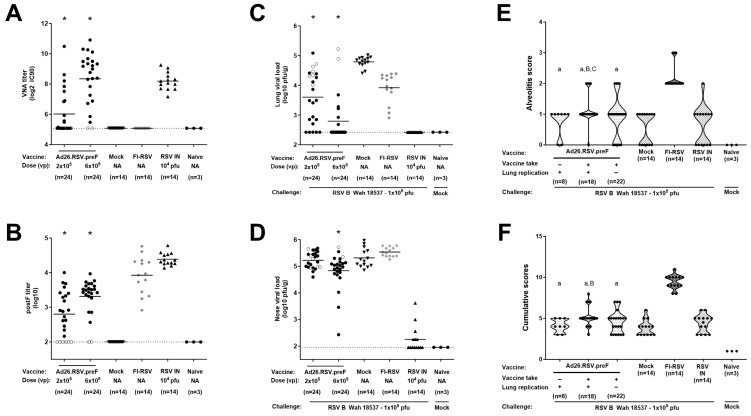
RSV B Wash breakthrough infection in the lower respiratory tract in animals immunized with suboptimal doses of Ad26.RSV.preF does not predispose to ERD. Cotton rats were immunized once with 2 × 10^5^ or 6 × 10^5^ vp Ad26.RSV.preF at day 0, whereas control groups received formulation buffer (mock), FI-RSV (at day 0 and day 28), or live RSV B Wash intranasally (RSV IN). On day 49, serum was collected. VNA titers against RSV CL57 (**A**) and F binding antibody titers (**B**) were determined. On day 49, animals were intranasally challenged with 1 × 10^5^ pfu RSV B Wash 18537. Naïve mock-challenged cotton rats are included as baseline control, indicated as “Naïve”. At 5 days post-challenge, RSV viral load was determined by plaque assay in homogenates from lung (**C**) or in nasal turbinates (**D**). Means are indicated with horizontal lines. LOD or LLoQ of the assays is indicated with dashed lines. (**E**) Histopathology scores for alveolitis were determined in H&E-stained lung sections. Ad26.RSV.preF immunized animals were subdivided based on detectable antibodies (vaccine take, positive in ELISA and/or VNAs) and detectable lung replication as determined by plaque assay (**E**). In addition, cumulative scores were calculated as the sum of the histopathology scores for alveolitis, peribronchiolitis, interstitial pneumonia, and perivasculitis (**F**). Scores of individual animals are shown with black dots, with open circles indicating individual animals without detectable antibody titers as determined by ELISA or VNAs in panels (**A**–**D**). Significant differences (adjusted *p* ≤ 0.05) in immunogenicity and efficacy readouts between Ad26.RSV.preF and mock immunized groups are indicated with asterisks (panels (**A**–**D**)). The frequency distribution is indicated by the shape of the violin plot in panels (**E**,**F**). Results from statistical analysis comparing the histopathology scores of the Ad26.RSV.preF immunized groups with the FI-RSV, mock immunized, or RSV intranasally pre-exposed control groups are indicated with A/a, B/b, or C/c, respectively, where *p* ≤ 0.05, with lowercase letters indicating significantly lower scores, and uppercase letters indicating significantly higher scores, compared to the respective control groups.

Therefore, the use of the heterologous RSV B Wash challenge model was further explored by lowering the vaccine dose to 2 × 10^5^ and 6 × 10^5^ vp. These low doses of Ad26.RSV.preF induced a wide range of RSV neutralizing and RSV F binding antibody titers, including 8 animals without detectable antibodies (indicated with open symbols in Figure 5). As expected, immunization with FI-RSV induced F-binding antibodies without neutralizing capacity, whereas the RSV intranasal pre-exposed group induced neutralizing as well as F-binding antibodies (Figure 5A,B). At 5 days post RSV B Wash challenge, lung and nose viral loads were assessed by plaque assay. For the 2 × 10^5^ dose group, 5 out of 24 animals had undetectable lung viral load, whereas others had detectable viral load, albeit mostly at a lower level compared with mock-immunized animals. For the 6 × 10^5^ vp dose, most animals (17 out of 24) had undetectable viral load, whereas 5 animals showed reduced viral load, and the 2 animals without detectable antibodies showed lung viral load comparable to the mock group (Figure 5C). Nose viral load was only reduced to a limited extent at these low vaccine doses (Figure 5D).

For the assessment of the pulmonary inflammatory parameters, we categorized the Ad26.RSV.preF-vaccinated animals in three subgroups: animals without detectable antibodies and with detectable viral replication in the lung (*n* = 8), animals with both detectable antibodies and detectable lung viral load (*n* = 18), and animals with detectable antibodies but absence of detectable lung viral load (*n* = 22). Alveolitis scores and cumulative scores for the subgroups of Ad26.RSV.preF-immunized animals were not significantly different from scores observed in mock-immunized and intranasal pre-exposed control groups, with the exception of scores in the group of animals with detectable antibodies and viral load that had marginally increased scores (*p* = 0.0135 and *p* = 0.0381 for alveolitis compared to mock or intranasal pre-exposed control groups, respectively; *p* = 0.0030 and *p* > 0.05 for cumulative scores compared to mock or intranasal pre-exposed control groups, respectively; Wilcoxon rank-sum test with 3-fold Bonferroni correction). However, the scores were highly significantly lower in all subgroups when compared with the FI-RSV control group for the parameters assessed (*p* < 0.0003) (Wilcoxon rank-sum test with 3-fold Bonferroni correction) (Figure 5E,F).

Taken together, these data show that, also after challenge with vaccine-heterologous RSV B Wash, there is no indication of predisposition to ERD after immunization with Ad26.RSV.preF, including in animals that fulfil the criteria for proper ERD assessment, i.e., adequate viral replication following RSV challenge, in the presence of vaccine-induced antibodies.

### 3.4. In Mice, Ad26.RSV.preF Dose-Dependently Induces Protection Against RSV A2, Without Any Signs of Predisposition to Enhanced Respiratory Disease

We additionally studied ERD predisposition using a mouse model, which is characterized by infiltration of eosinophils and mucus secretion, in addition to the induction of alveolitis, peribronchiolitis, interstitial pneumonia, and perivasculitis.

**Figure 6:**
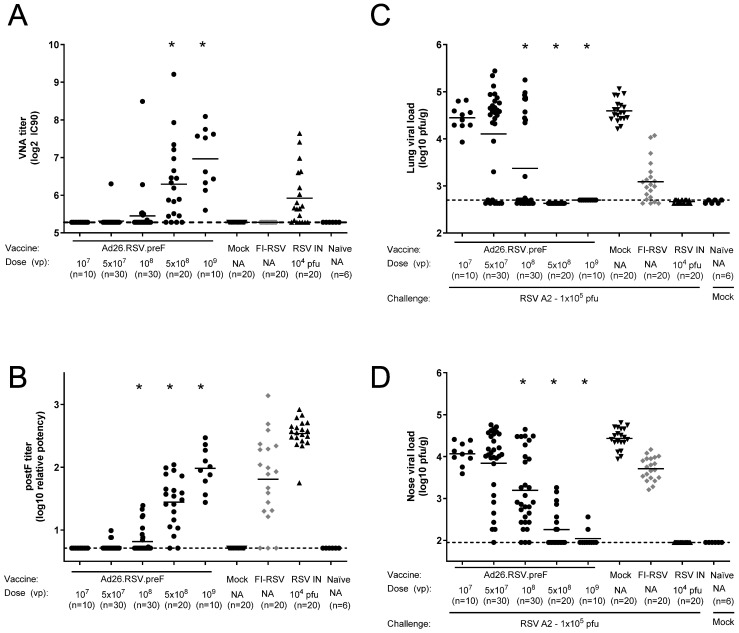
Ad26.RSV.preF induces highly neutralizing antibodies and dose-dependently protects mice against challenge with RSV A2. Mice were immunized with increasing doses of Ad26.RSV.preF, whereas control groups received formulation buffer (mock), FI-RSV, or live RSV intranasally. On day 49, after immunization, pre-challenge serum was collected, and animals received an intranasal challenge with RSV A2 (1 × 10^5^ pfu). Pre-challenge VNA titers against RSV CL57 (**A**) and F binding antibody titers (**B**). At 5 days post-challenge, RSV viral load was determined by plaque assay in homogenates of lung (**C**) or nasal turbinates (**D**). LOD or LLoQ of the assays are indicated with dashed lines, and means are indicated with horizontal lines. Data presented are a combination of results from two independent challenge experiments. Significant differences (adjusted *p* ≤ 0.05) between Ad26.RSV.preF and mock-immunized groups are indicated with asterisks.

Balb/c mice were immunized with a single dose of Ad26.RSV.preF in a range of 10^7^ up to 10^9^ vp, and control groups received formulation buffer (mock), two immunizations of FI-RSV, or an intranasal RSV A2 pre-exposure. Prior to challenge, at day 49, the Ad26.RSV.preF immunized mice showed a dose-dependent induction of RSV neutralizing and F binding antibodies (Figure 6A,B). In accordance with the observations for the cotton rat model, mice immunized with FI-RSV showed F binding titers without neutralizing capacity. Lung and nose viral loads assessed 5 days post-challenge showed vaccine dose-dependent significant reduction in viral replication, with full protection in the lung (Figure 6C) and clear reduction in nose viral load at 5 × 10^8^ and 1 × 10^9^ vp Ad26.RSV.preF doses (Figure 6D).

**Figure 7:**
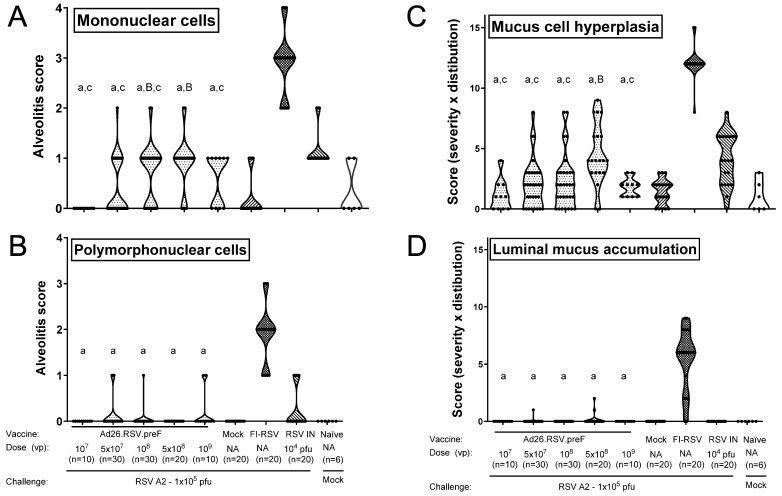
Ad26.RSV.preF does not predispose to ERD in the mouse RSV A2 challenge model. Mice were immunized with increasing doses of Ad26.RSV.preF, whereas control groups received formulation buffer (mock), FI-RSV, or live RSV intranasally. At 49 days post-immunization, animals were intranasally challenged with RSV A2 (1 × 10^5^ pfu). Sections were made from lung tissue isolated at day 5 post-challenge. Histological analysis was performed on H&E-stained slides for alveolitis on a scoring scale from 0 to 4, thereby scoring infiltrates of mononuclear (**A**) and polymorphonuclear cells (**B**) separately. PAS-stained slides were scored for mucus cell hyperplasia (proliferation and enlargement of mucous/goblet cells) (**C**) or luminal mucus accumulation (bronchiolar luminal containment of mucus, exudates of inflammatory cells, and cell debris) (**D**). Parameters were scored for severity and distribution, both on a scale from 0 to 4, and the product of both parameters is shown. Scores of individual animals are shown with black dots, and the frequency distribution is indicated by the shape of the violin plot. Results from statistical analysis comparing the histopathology scores of the Ad26.RSV.preF-immunized groups with the FI-RSV, mock-immunized, or RSV intranasally pre-exposed control groups are indicated with A/a, B/b, or C/c, respectively, where *p* ≤ 0.05, with lowercase letters indicating significantly lower scores and uppercase letters indicating significantly higher scores compared to the respective control groups. Data presented are a combination of results from two independent challenge experiments.

Histopathological alveolitis scores in the lungs were determined separately for infiltrates of mononuclear cells and polymorphonuclear (PMN) cells. Whereas high scores for alveolitis with infiltrates of mononuclear cells were observed in FI-RSV-immunized mice, these scores were significantly lower for all doses of the Ad26.RSV.preF vaccine (*p* < 0.0005) (Wilcoxon rank-sum test with 5-fold Bonferroni correction), which were similar to or significantly lower than in the intranasal pre-exposed group (Figure 7A). All FI-RSV-immunized mice showed alveolar PMN infiltrates (scores 1 to 3). In contrast, only 7 out of 100 Ad26.RSV.preF-immunized mice had PMN alveolitis, with a score of 1 (Figure 7B).

Lung sections were additionally stained with Periodic Acid-Schiff (PAS) for scoring of mucus cell hyperplasia and luminal mucus accumulation. Representative examples of PAS-stained sections of vaccine and control groups are shown in Figure S3. Mucus cell hyperplasia scores of the Ad26.RSV.preF immunized mice were significantly lower than the RSV intranasally pre-exposed animals (*p* ≤ 0.0200) (Wilcoxon rank-sum test with 5-fold Bonferroni correction) except for the group immunized with the 5 × 10^8^ vp dose level. The mucus cell hyperplasia scores of the Ad26.RSV.preF-immunized mice were also significantly lower compared with the FI-RSV-immunized animals (*p* < 0.0005) (Wilcoxon rank-sum test with 5-fold Bonferroni correction) (Figure 7C). In addition, almost all FI-RSV-immunized animals showed substantial luminal mucus accumulation in the lung (median score 6), whereas mucus accumulation was mostly absent in the Ad26.RSV.preF-immunized animals and RSV intranasally pre-exposed animals (Figure 7D). Altogether, the mouse model also does not indicate ERD predisposition after immunization with Ad26.RSV.preF. Of note, of the one hundred Ad26.RSV.preF-immunized mice analyzed, three animals showed lung viral load in the presence of detectable antibodies (Table S1). Histopathology scores of those three animals were identical to the rest of the group.

## 4. Discussion

Here we have shown the results of a series of cotton rat and mouse studies that preclinically assessed predisposition to ERD of the Ad26.RSV.preF vaccine that is intended for the development of a pediatric RSV vaccine. None of these studies indicated any signs of predisposition to ERD, irrespective of the vaccine dose used, presence of detectable lung viral replication, or the virus strain used for challenge. For an overview of the total number of animals analyzed, see Table S1. Pathological scores for Ad26.RSV.preF-vaccinated animals were lower than for FI-RSV-vaccinated animals and comparable to or lower than for animals intranasally pre-exposed to RSV prior to challenge to mimic natural repeated infection. Based on these data, we concluded that there are no signs of predisposition to ERD after immunization with Ad26.RSV.preF, reducing the risk for further clinical development of Ad26.RSV.preF for the pediatric population.

The clinical opportunities to prevent RSV disease in young infants have been substantially expanded by the option to induce passive immunity in infants via administration of long-acting monoclonal antibodies or via maternal vaccination with preF protein [26]. However, there is still a need for approaches employing active vaccination as well. Firstly, passively acquired antibodies wane over time, thereby only providing protection during a single RSV season. Whereas the disease burden is highest in children under 6 months of age, substantial clinically relevant RSV disease is observed in older age categories as well [27]. Secondly, the consequences of RSV infection in low-middle-income countries are particularly devastating, and the currently available immunoprophylaxis is costly and has practical logistic delivery challenges. The maternal vaccination program has not been widely introduced yet, and current vaccination rates are relatively low. Lastly, data on the influence of immune prophylaxis or maternal immunization on the response to secondary RSV infections is scarce. Recently, exacerbation of lung inflammation after secondary RSV infection after maternal immunization was shown in a mouse model, although the offspring showed complete protection against primary infection [28]. Therefore, active infant vaccination programs can offer a durable and cost-effective alternative to existing clinical options.

For protein-based vaccines and inactivated virus vaccines, the theoretical risk for ERD can be considered relatively high, as in the vaccine recipient, there is a lack of intracellular expression and processing needed for proper induction of a Th1 response associated with induction of RSV-specific CD8+ cytotoxic T lymphocytes [15]. Indeed, it was demonstrated earlier that preF and postF protein vaccines predisposed to vaccine-enhanced disease in cotton rats, only when given at low doses [14]. Interestingly, this was observed with both Th1- and Th2-skewing adjuvants, implying that Th1 skewing per se does not abrogate the risk for ERD. At the low doses used in those studies, the preF protein did induce neutralizing antibodies, in combination with partial to full reduction in viral replication in the lungs. This demonstrates that ERD can be observed in preclinical models in the absence of lung viral replication when assessed by plaque assay, which had been suggested to impede proper ERD assessment [15]. The published findings, however, underscore the importance of ERD assessment using a wide vaccine dose range, including suboptimal doses that do not provide full protection from infection. Live attenuated vaccines and vaccines based on viral vectors can be considered less likely to predispose to the induction of ERD. Indeed, live attenuated virus vaccines given intranasally have been evaluated in RSV-seronegative infants without ERD-related safety findings [29]. We have shown earlier that Ad26.RSV.preF induced a favorable immune response in preclinical models, with a Th1 immune profile associated with good CD8+ T-cell responses [17,22]. In a more extensive analysis, presented in this manuscript, we did not detect any signs of ERD in a large number of animals analyzed, where the viral vector was tested in a large dose range, including sub-protective doses, in combination with different challenge viruses.

In general, studies in animal models can indicate a risk for vaccine-induced ERD; however, the absence of any markers of predisposition to ERD, as presented in this paper, cannot entirely rule out the risk for ERD in infants. In addition, caution should be taken with direct extrapolation of data generated in adult animals to the risk for ERD in infants. The relative immaturity of the lungs as well as the immune system of infants might have contributed to the development of ERD, which might not be fully captured when using adult animal models. During an advisory meeting on ERD of the Vaccines and Related Biological Products Advisory Committee (VRBPAC) held in 2017, it was discussed that preclinical animal testing comprises the first important step for mitigating the risk of ERD. A second step should include the generation of clinical safety and immunogenicity data in adults and RSV-experienced children and infants. And the final step consists of a careful strategy of ERD assessment in RSV-naïve infants, including focused eligibility criteria for inclusion, age de-escalation, and prolonged duration of follow-up through two RSV seasons [15]. While preclinical ERD assessment for Ad26.RSV.preF is presented in this manuscript, and favorable clinical safety and immunogenicity data have been described for the elderly [18], as well as for adults and RSV-seropositive children of 12–24 months of age [20]. Results of a phase 1/2A study in a small cohort of RSV-seronegative children of 12–24 months of age were published recently and demonstrated a favorable safety and immunogenicity profile of the Ad26.RSV.preF vector, without indication of ERD predisposition [21]. In contrast, a clinical study in RSV-negative toddlers of 5 to 8 months of age immunized with an mRNA-encoded RSV preF vaccine was paused due to an imbalance in the number of severe RSV cases (https://www.fda.gov/media/184301/download, accessed on 28 October 2025). The limited preclinical data for this mRNA-based vaccine available in the public domain did not indicate a risk for ERD [30], but it is too incomplete to allow a conclusion on the value of the ERD cotton rat model used. Although the significance of this clinical finding is unclear due to the low numbers of study participants, this emphasizes the importance of a careful age de-escalation approach, even for vaccines with demonstrated favorable immunogenicity profiles and absence of ERD features in preclinical models.

## 5. Conclusions

As there is still a need for active immunization approaches for protecting young infants from respiratory illness induced by RSV, the preclinical results presented in this manuscript showing no signs of ERD after Ad26.RSV.preF immunization were essential for de-risking the pediatric development of this vaccine to conduct the first study in RSV-seronegative children.

## Data Availability

All data to understand and assess the conclusions of this research are available in the main text and Supplemental Materials. The raw data that support the findings of this study are available from the corresponding author upon reasonable request.

## Supplementary Materials

The following supporting information can be downloaded at: https://www.mdpi.com/article/10.3390/vaccines14010087/s1, Figure S1: Examples of histological pictures of H&E-stained sections; Figure S2: Low doses of Ad26.RSV.preF do not induce histopathological signs of predisposition to ERD, even at higher RSV A2 challenge doses; Figure S3: Examples of histological pictures of PAS stained section of mouse lung tissue; Table S1: Overview of total number of animals analyzed for histopathological features of ERD.
