# Supplementary material for: Adenovector 26 Encoded RSV Prefusion F Protein (Ad26.RSV.preF) Does Not Predispose to Enhanced Respiratory Disease in Preclinical Rodent Models
Source: Vaccines (Basel). 2026 Jan 15;14(1):87. doi: 10.3390/vaccines14010087 (PMC12846498; doi:10.3390/vaccines14010087)
Supplement: Supplementary file 1 [file vaccines-14-00087-s001.zip › vaccines-4027905-supplementary.pdf]

Supplementary materials, accompanying "Adenovector 26 encoded RSV prefusion F protein (Ad26.RSV.preF) does not predispose to enhanced respiratory disease in preclinical rodent models" by Renske Bolder et al.

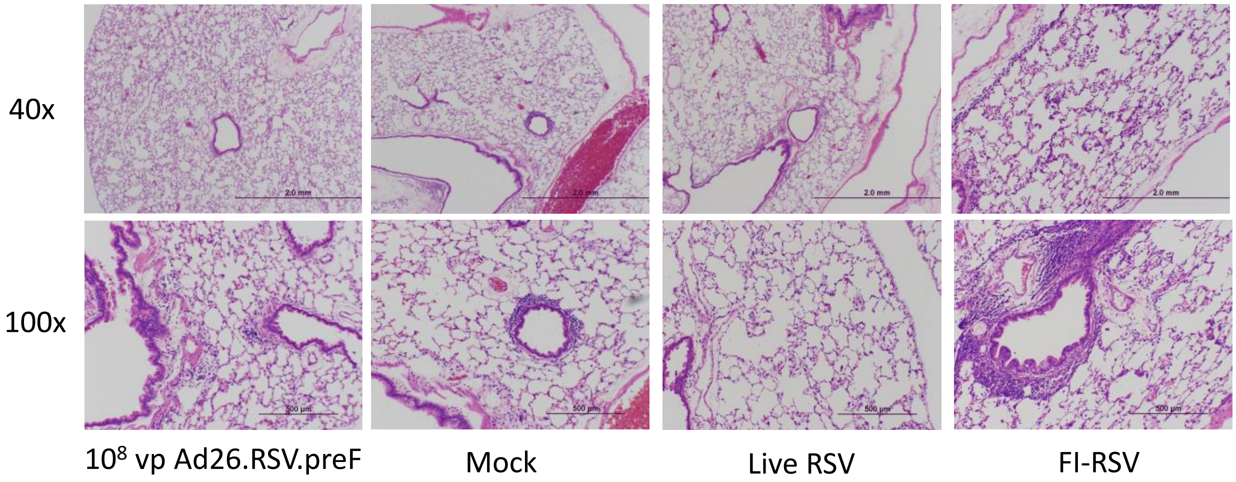

Figure S1: Examples of histological pictures of H&E-stained sections. Cotton rats were immunized at day 0 with 10^8 vp Ad26.RSV.preF, formulation buffer (mock), live RSV A2 intranasally or FI-RSV (day 0 and day 28). At day 49, animals were intranasally challenged with RSV A2 ($1x10^5$ pfu). Sections were made from lung tissue isolated at day 5 post challenge, and stained with H&E. Representative examples are shown at 40x or 100x magnification.

Supplementary materials, accompanying "Adenovector 26 encoded RSV prefusion F protein (Ad26.RSV.preF) does not predispose to enhanced respiratory disease in preclinical rodent models" by Renske Bolder et al.

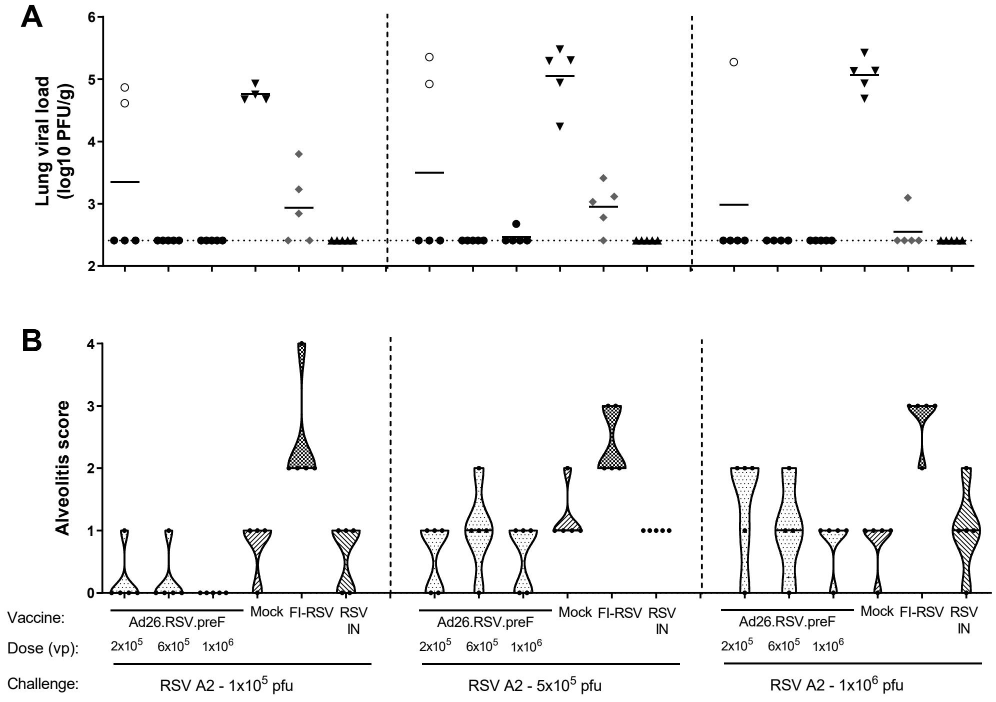

Figure S2: Low doses of Ad26.RSV.preF do not induce histopathological signs of predisposition to ERD, even at higher RSV A2 challenge doses. Cotton rats were immunized with $2x10^5$, $6x10^5$ or $1x10^6$ vp Ad26.RSV.preF at day 0, whereas control groups received formulation buffer (mock), FI-RSV (day 0 and day 28) or live RSV A2 intranasally. At day 49, animals were intranasally challenged with increasing doses of RSV A2, $1x10^5$ pfu (left panels), $5x10^5$ pfu (middle panels) and $1x10^6$ pfu (right panels). (**A**) At 5 days post challenge, RSV viral load was determined in lung homogenates by plaque assay. Scores of individual animals are shown with black or grey symbols, with open circles indicating individual animals without detectable antibody titers as determined by ELISA or VNA assays. Means are indicated with horizontal lines. LOD of the plaque assay is indicated with dotted lines. (**B**) At 5 days post challenge, histopathology scores for alveolitis were determined in H&E-stained lung sections. Scores of individual animals are shown with black dots, with the frequency distribution indicated by the shape of the violin plot.

Supplementary materials, accompanying "Adenovector 26 encoded RSV prefusion F protein (Ad26.RSV.preF) does not predispose to enhanced respiratory disease in preclinical rodent models" by Renske Bolder et al.

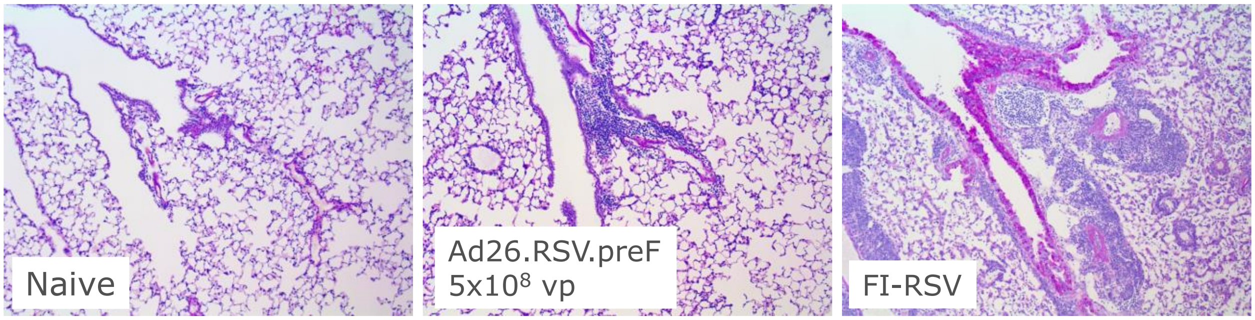

Figure S3: Examples of histological pictures of PAS stained section of mouse lung tissue.

Mice were immunized at day 0 with 5x10^8 vp Ad26.RSV.preF, formulation buffer, or FI-RSV (day 0 and day 28). At day 49, animals were intranasally challenged with RSV A2 (1x10^5 pfu), or left unchallenged (indicated with Naïve). Sections were made from lung tissue isolated at day 5 post challenge, and stained with PAS. Representative examples are shown at 10x magnification.

Supplementary materials, accompanying "Adenovector 26 encoded RSV prefusion F protein (Ad26.RSV.preF) does not predispose to enhanced respiratory disease in preclinical rodent models" by Renske Bolder et al.

		**Species:**	Cotton rat	Cotton rat	Cotton rat	Mouse
		**Challenge strain:**	RSV A2	RSV A2	RSV B Wash 18537	RSV A2
		**Challenge dose (pfu):**	$1{\times}10^5$	$5{\times}10^5$ - $1{\times}10^6$	$1{\times}10^5$	$1{\times}10^5$
		**Ad26.RSV.preF dose (vp):**	$1{\times}10^5$- $2{\times}10^{10}$	$2{\times}10^5$- $1{\times}10^6$	$2{\times}10^5$- $1{\times}10^8$	$5{\times}10^7$- $1{\times}10^9$
**Treatment**	**Vaccine Take** [b]	**Lung Viral Load**	**5 studies #animals**	**1 study #animals**	**2 studies #animals**	**2 studies #animals**
Mock			34	10	21	20
RSV IN			37	10	21	20
FI-RSV			35	10	21	20
Ad26.RSV.preF	Yes	No	118/114[a]	0	42	51
Ad26.RSV.preF	Yes	Yes (PCR)	29[c]	25	ND	ND
Ad26.RSV.preF	Yes	Yes (Plaque)	1	1	19	3
Ad26.RSV.preF	No	Yes (Plaque)	20	3	8	41
Ad26.RSV.preF	No	No	0	0	0	5
**Ad26.RSV.preF**	**Total**		**168/164[a]**	**29**	**69**	**100**

[a] N dependent on the parameter measured
[b] defined as VNA and/or F binding antibody titers above background
[c] PCR was only performed in 2 out of 5 studies
ND: not determined, PCR was not performed

Table S1: Overview of total number of animals analyzed for histopathological features of ERD. Overview of the number of cotton rats and mice that were analyzed by histopathology for signs of ERD, categorized based on proved vaccine take (VNA and/or F binding antibody titers above background after immunization with Ad26.RSV.preF) and lung viral load, determined by plaque assay or RT-qPCR. Note, no signs of ERD have been observed in any of the Ad26.RSV.preF immunized subgroups, whereas FI-RSV immunized animals consistently showed histopathological changes associated with ERD.
